# Trends in breast, ovarian and cervical cancer incidence in Mumbai, India over a 30-year period, 1976–2005: an age–period–cohort analysis

**DOI:** 10.1038/bjc.2011.301

**Published:** 2011-08-09

**Authors:** P K Dhillon, B B Yeole, R Dikshit, A P Kurkure, F Bray

**Affiliations:** 1 Mumbai Cancer Registry, Indian Cancer Society, Mumbai, India; 2 South Asia Network for Chronic Disease, Public Health Foundation of India, C-1/52 Safdarjung Development Area, First Floor, New Delhi, 110016, India; 3 Department of Epidemiology, Tata Memorial Hospital, Mumbai, India; 4 Section of Cancer Information, International Agency for Research on Cancer, Lyon, France

**Keywords:** neoplasm, breast, ovary, cervix, time trends, India

## Abstract

**Background:**

Demographic, socioeconomic and cultural changes in India have increased longevity, delayed childbearing, decreased parity and resulted in a more westernised lifestyle, contributing to the increasing burden of cancer, especially among women.

**Methods:**

We evaluated secular changes in the incidence of breast, cervical and ovarian cancer in Mumbai women aged 30–64 between 1976 and 2005. Age-standardised incidence rates were calculated and presented by site and calendar period. An age–period–cohort (APC) analysis quantified recent time trends and the significance of birth cohort and calendar period effects. The estimated annual percent change (EAPC) was obtained from the drift parameter, expressing the linear time trend common to both calendar period and birth cohort.

**Results:**

Over the 30-year study period, the age-standardised rates significantly increased for breast cancer (EAPC: 1.1% (95% confidence interval (CI): 1.0, 1.3)), significantly decreased for cervical cancer (EAPC: −1.8% (95% CI: −2.0, −1.6)) and there was no statistically significant change for ovarian cancer (EAPC: 0.3% (95% CI: −0.1, 0.6)). For breast and cervical cancer, the best-fitting model was the APC model.

**Conclusions:**

The rates of breast, cervical and ovarian cancer remain low in comparison with western countries, and the divergent trends of breast (increasing) and cervical cancer (decreasing) in Mumbai were similar to those observed in several other Asian countries. The changing risk profile in successive generations – improved education, higher socioeconomic status, later age at marriage and at first child, and lower parity – may in combination partially explain the diverging generational changes in breast and cervical cancer in Mumbai in the last decades.

The increasing incidence of cancer in India has mirrored trends in developed countries, although the rates for major sites, such as female breast cancer have remained comparatively low. Over the last two decades, the urbanisation and increasing modernisation of India has transformed education, lifestyle, health-care access and longevity, and has contributed to an increased risk profile for chronic diseases such as cancer (Reddy, 1993, 2007; Murray and Lopez, 1996; Ghaffar *et al*, 2004). In the large metropolitan cities of India, such as Mumbai (formerly Bombay), these transitions are apparent in women, for whom the pursuit of higher education, entry into the workforce, delayed childbearing and lower parity are characteristic of those in more recent generations (National Family Health Survey India (NFHS-1) 1992–93: Maharashtra, India, 1995; National Family Health Survey India (NFHS-2): 1998–99 Maharashtra, India, 2000). A higher per capita increase has also paralleled a shift in dietary practices towards a greater consumption of animal products and total fat, (World Bank. World development report, 1993; Shetty, 2002) more sedentary lifestyles and increasing prevalence of overweight and obesity in higher social classes of Indian cities, (Singh *et al*, 1999; Vaz and Bharathi, 2000; Gupta *et al*, 2003) estimated at 29.7% in a recent survey of Mumbai women (Shukla *et al*, 2002).

This study aims to quantify the recent time trends in the most commonly occurring female cancers in Mumbai women, with a view to better understanding the contribution of changing lifestyle. An age–period–cohort (APC) analysis of breast, ovarian and cervical cancer incidence trends was conducted, using the population-based cancer registry data in Mumbai, India over a 30-year period, 1976–2005.

## Materials and methods

### Incidence

All incident cases of breast, ovarian and cervical cancer (ICD-10 C50, C53 and C56, respectively) diagnosed in 1976–2005 were extracted from the Mumbai Cancer Registry. Registry personnel conducted routine visits to 140 collaborating hospitals over a 603 square kilometre area of over 12 million residents to review pathology logs on new cancer diagnoses. Medical charts and pathology reports were reviewed for clinical information including date and method of diagnosis, histological grade and subtype, and stage of disease as well as demographic information such as age, gender, ethnicity and marital status. The Mumbai Registry data set has consistently met IARC's quality standards with respect to inclusion in consecutive volumes of their Cancer Incidence of Five Continents series (Parkin *et al*, 2005; Curado *et al*, 2007).

The study population included women aged 30–64 years of age who were residents of Mumbai between 1976 and 2005. Annual population estimates were interpolated from decennial Census of India data (Census of India: Census population tables Maharashtra part IIC 1981; Census of India: Census population tables. Maharashtra part IIA 1991; Census of India: Series 28, Maharashtra provisional population 2001). Subjects were categorised into seven 5-year age groups (30–34, 35–39…60–64) and six 5-year calendar periods (1976–1980, 1981–1985…2001–2005) based on their respective age and year of diagnosis. Twelve 10-year synthetic birth cohorts (1911–1920, 1921–1930…1966–1975) were obtained by subtracting the midpoint of 5-year age band from the central year of the 5-year period of diagnosis.

### Statistical analyses

Age-standardised incidence rates truncated for the age group 30–64 were calculated using the Segi/Doll world standard (Segi and Kurihara, 1960; Doll *et al*, 1966) by cancer site and calendar period. Graphical descriptions of the age-specific incidence rates according to cohort and period are shown for each age group on a semi-log scale. Relatively parallel lines on the period (cohort) scale of these graphs are indicative of the importance of period (cohort) effects. Period effects denote systematic changes that affect rates in all study age groups at a given point in time, often representing artefacts such as changes in completeness of registration, diagnostic practices or disease classification. They can, however, occur through the introduction of specific environmental factors to which all population members are exposed irrespective of age, or as a result of interventions such as screening that affect all age groups at certain point in calendar time. Cohort effects tend to reflect changes in exposure to the risk factors that are in operation in successive generations; these may relate to birth itself or approximate factors related to birth by exerting influences that are shared in the same group as they age together.

A more formal assessment of the contribution of age, period and cohort effects involved the fitting of APC models to the trends (Clayton and Schifflers, 1987a, 1987b). Overall goodness-of-fit tests as well as statistical tests for the contribution of the overall slope (*net drift*) and the effects of period and cohort curvature, were obtained using the analysis of deviance of nested models, as suggested by Clayton and Schifflers (1987b). In this framework, the importance of non-linear period and cohort effects is statistically tested on comparison with the simple trend model (age-drift model) as well as the two-factor (age–period and age–cohort) models. The final model was selected on the grounds of parsimony, using the log-likelihood ratio test to evaluate nested models on the basis of the addition of cohort and period terms. We used results from the age-drift model to summarise the magnitude and direction of temporal trends over the period 1991–2005. The net drift parameter is a one-degree-of-freedom linear term for time that represents the estimated annual percent change (EAPC) in the rates over the passage of time that is common to calendar period and birth cohort (Clayton and Schifflers, 1987a). The EAPC is the average change in the trend over the designated time of study and is linear on a log scale and thus comparable irrespective of the magnitude of the rates at baseline.

Finally, the APC model analysis included a presentation of the model effects on assuming a period slope of zero, that is, allocating the drift to birth cohort (Holford, 1991). This is an arbitrary assignment to obtain a unique solution, and must be interpreted with considerable caution. Nevertheless, it is not without a certain rationale given an *a priori* hypothesis that the increasing trends in breast and ovarian cancer, for example, are allied to generational influences; a changing prevalence and distribution of known and unknown lifestyle and environmental factors in the female population of Mumbai, should, given a sufficient time lag, result in changing cancer rates observed in successive birth cohorts. Stata (StataCorp., 2007) and R (R Development Core Team, 2008) were used for data management and analysis.

## Results

Breast and cervical cancer were the most frequent cancers occurring in Mumbai women and together with ovarian cancer, accounted for more than half of all female cancers in the study period (Table 1). The rates of breast cancer among women aged 30–64 have risen gradually over the 30-year study period, with the mean increase estimated at 1.1% per year, and representing 32% of the female cancer burden in 2001–2005. In contrast, cervical cancer rates among women in the same age range decreased by 1.8% per year on average but still represents 16% of the total female cancer burden in the latest 5-year period. The age-standardised incidence of ovarian cancer among 30- to 64-year old women were reasonably stable overall, with the proportion of total female cancer incidence remaining at 7% over time.

### Breast cancer

**Figure 1:**
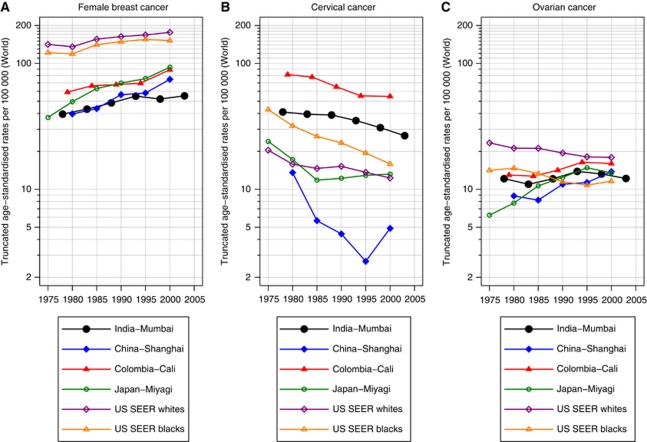
Comparison of time trends of truncated (30–64 years) age-standardised (world) rates of (**A**) female breast cancer; (**B**) cervical cancer; (**C**) ovarian cancer in Mumbai females 1976–2005, *vs* selected populations worldwide 1973–2002, extracted from successive volumes of Cancer Incidence in Five Continents.

**Figure 2:**
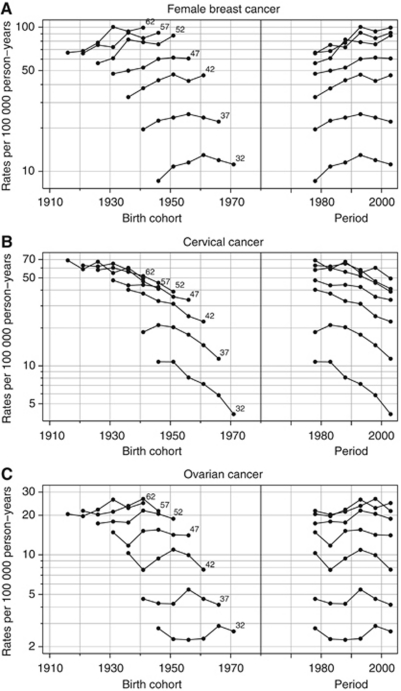
Observed rates of (**A**) female breast cancer; (**B**) cervical cancer; (**C**) ovarian cancer in Mumbai females aged 30–64 and diagnosed 1976–2005. Rates are plotted *vs* calendar period and birth cohort for each age at diagnosis group.

**Figure 3:**
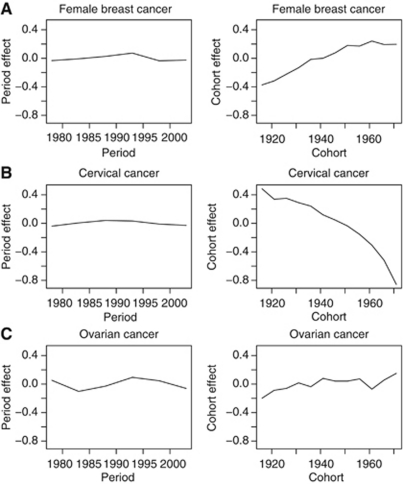
Graphical representation of parameters from the full APC models of (**A**) female breast cancer, (**B**) cervical cancer and (**C**) ovarian cancer of Mumbai females aged 30–64 and diagnosed 1976–2005 on specifying a period slope of zero, thereby allowing a unique (but necessarily somewhat arbitrary) solution.

The age-standardised female breast cancer rates for women in Mumbai were consistently lower than women of other medium-resource countries (Figure 1A), despite the significant average increases over the 30-year period of 1.1% per annum (Table 1). The changes in rates were smaller within the latest 15-year period. The magnitude and change of incidence rates over time were similar to women in Shanghai, China and *ca* 2000, rates in Mumbai were one-third of those seen in white women diagnosed with breast cancer in the United States (Figure 1A). The respective graphs of rates by calendar period and birth cohort indicate lower age-specific rates for women diagnosed in earlier time periods and for those born in earlier cohorts (Figure 2A). The close-to parallel lines exhibited between successive birth cohorts and periods of diagnosis convey little with regards the relative importance of cohort and period curvature, although there is a point of deflection downwards across all ages in the period 1996–2000 (except 45–49 year olds, where there was a slight increase) followed by an increase restricted to older women in the last period. The APC model analyses in Tables 2 indicates that non-linear period and cohort effects were both significant, yielding the full APC model as the best fitting for breast cancer trends in Mumbai women. In Figure 3A, the drift is added to the non-linear birth cohort effects, and therefore conveys the rather linear increases in successive generations as well as the period curvature resulting from small declines in the last decade, especially in younger women (age <50 years).

### Cervical cancer

The trend for cervical cancer among Mumbai women was consistent with a general pattern of declines in many populations worldwide (Figure 1B). The age-specific rates decreased for all women aged 30–64 (1.8% decline overall, Table 1) with the downwards trends more striking – a mean decline of 2.8% per annum – in the most recent period 1991–2005 (Table 1) and for women under the age of 50 years (Figure 2B), and the greatest changes observed among younger women (Table 2). The analysis of deviance indicated a significant lack-of-fit of the full APC model and significant non-linear effects for both birth cohort and calendar period. The downward concavity in the period effects as shown in Figure 3 suggests a slight deceleration in the rate of decline over time, and the APC analysis shows it is weaker than the cohort effect (Table 2).

### Ovarian cancer

The absolute and relative change in Mumbai's ovarian cancer rates over time was minor compared with other countries overall (Figure 1C). The rates in Mumbai remained intermediate between that of Cali, Colombia and Shanghai, China women throughout the 30-year period, while female rates in Miyagi, Japan overtook Mumbai women by the late 1990s from a baseline rate one-third that of Mumbai 1976–1980. In Mumbai, the rates fluctuated between an increasing trend up to the mid-1990s to slight decreases thereafter (Figure 1C). We also observed fluctuations in the trend by age group (Figure 2C). There was no significant change in rates over time, and the analysis of deviance indicated that the age–period model was the most parsimonious APC model. The significant non-linear period effect (Table 2) may have resulted from a minor decline within the last three calendar periods, 1991–2005 (Figure 2C).

## Discussion

This study has shown that the evolution of breast, cervical and ovarian cancer rates over 30 years in Mumbai, India is similar to the population trends in several other Asian and low-to-medium-resource countries. The annual rate of breast cancer significantly increased during the period 1976 and 2005, while cervical cancer rates significantly decreased. For all three sites, secular changes at the population level need to be examined in terms of the possible screening interventions and/or changing diagnostic patterns (as possible period effects) against a changing prevalence and distribution of risk factors, which may show up as changes in rates among successive generations (cohort effects).

Age–period–cohort analyses of secular changes in breast cancer have yielded different temporal patterns for western *vs* Asian countries (Seow *et al*, 1996; Leung *et al*, 2002; Li and Daling, 2007). In the United States and Canada, breast cancer increased in the 1980s and 1990s, and began to plateau in the late 1990s, (Li *et al*, 2003; Althuis *et al*, 2005), most likely due to a saturation of mammography screening (Li and Daling, 2007), which suggests stronger period effects. In Asian countries, where rates are lower and trends tend to be still increasing, tumours are predominantly detected by physical examination (with the exception of Japan (Shapiro *et al*, 1998)). Studies in the region have tended to attribute the increase to cohort effects (Seow *et al*, 1996; Leung *et al*, 2002) and a general westernisation effect that may include changes in dietary and fertility patterns alongside an increasingly affluent and sedentary lifestyle (Jin *et al*, 1999; Yip *et al*, 2001; Leung *et al*, 2002).

For ovarian cancer, the trends vary according to geographic region – with decreasing rates in the United States and northern Europe (Kjaerbye-Thygesen *et al*, 2005; Bray *et al* 2005c; Morris *et al*, 2008) but increasing rates in a few southern and eastern European countries and in Asian countries including Japan, China and Hong Kong (Marugame and Hirabayashi, 2007; Song *et al*, 2008). For cervical cancer and squamous cell carcinoma specifically, the time trends are more consistent worldwide, with systematic decreases since the 1980s or 1990s (Levi *et al*, 1994; Walker *et al*, 1998; Liu *et al*, 2001; Siesling *et al*, 2008) attributable to effective screening programmes and changing sexual behaviour allied to the acquisition and persistent infection of high-risk HPV types (Minami *et al*, 1996; Bergstrom *et al*, 1999; Liu *et al*, 2001; Taylor *et al*, 2001; Bray *et al*, 2005a). The incidence of cervical adenocarcinoma in our population was too low for APC modelling, but we did find a slight increase in the proportion of adenocarcinomas from 4.7% of all cervical cancers in 1976 to 5.4% in 2000 (data not shown), which is also consistent with the increasing trend observed by others in developed countries (Visioli *et al*, 2004; Wang *et al*, 2004; Bulk *et al*, 2005; Bray *et al*, 2005b).

On the surface, a limitation of this study is the lower proportion of microscopically confirmed cases, as compared with many Western registries. In 1993–1997, the Mumbai cancer registry had histological verification for 84%, 84% and 77% of breast, cervical and ovarian cancer cases, respectively (Yeole, 2001). A relatively low proportion of morphological verification is in part, however, the result of highly qualified medical personnel using radiological and other less costly evidence for determining cancer diagnoses; cases more often present with metastatic disease. Data in this medium-resource setting have, however, been shown to be reasonably reliable and complete (Yeole and Jussawalla, 1988), and the registry data have met the criteria for inclusion in successive volumes of IARC's Cancer Incidence in Five Continents (IARC) publications.

A limitation to using APC modelling is that the models do not account for *linear* generational changes in the rates and given the complexity of trends, the APC models may therefore be considered a rather blunt instrument for detecting non-linear effects only. However, the models detected significant cohort and period effects for both cervical and breast cancer, where clear trends were observed. For ovarian cancer, where there was no clear overall trend, the APC model still yielded a significant period effect, explaining the consistent decrease observed across all the age groups in the last period (Figure 2C).

The relative straightforwardness of fitting APC models is at odds with the difficulties in providing an informed presentation of the model parameters, given the irresolvable issue of non-identifiability. One further linear constraint must be imposed to ensure a unique solution, but the crux of the problem is that the choice of model constraint and the resulting parameter estimates are completely arbitrary in the absence of compelling external information that one can bring to bear in making the selection. We have assumed generational influences predominate for all three cancers and that they reflect a changing prevalence and distribution of risk factors in the female population of Mumbai. While a non-zero period slope for some of these trends cannot be entirely dismissed – for example, via changing levels of case ascertainment – it is unlikely to explain the long-term increases in the regular trend.

Another limitation of this study is its ecological approach, which prevents causal inferences of associations between observed trends at the population level and risk factors at the individual level. However, these analyses are an effective and resource-efficient method for evaluating temporal trends at the population level and whether observed changes might reflect data artefacts, interventions or a true underlying change in risk, a critical step before more costly analytic studies of putative risk factors in various Indian communities are considered.

### Breast cancer

The APC models yielded significant cohort and period effects for breast cancer, suggesting that underlying risk factor patterns as well as changes in awareness, screening and/or diagnostic procedures may explain the significant increasing trends for breast cancer risk in Mumbai women over the 30-year study period. Increasing rates may be attributed to later age at first birth and lower total parity of more recent generations (National Family Health Survey India (NFHS-1) 1992–93: Maharastra, India, 1995; National Family Health Survey India (NFHS-2): 1998–99 Maharastra, India, 2000). In a case series of 11780 tumours (Shet *et al*, 2009) from the largest tertiary care referral centre for the Mumbai Cancer Registry, the investigators reported a redistribution of hormone receptor expression over an 8-year period – ER+ (7.5–10.6%) and ER+/PR+ status (25 to 41.8%) increased between 1999 and 2006 while PR+ decreased (21–3.4%). This lends support to the hypothesis that changing reproductive factors may have a role in the observed trends. Although there is no population-based organised breast cancer screening programme for women in the region, the increasing use of mammography and a heightened awareness among physicians and patients led to improvements in the clinical extent of disease at diagnosis over time. Among newly diagnosed cases, a third presented with localised disease in 1976 (33.8%) while nearly half had localised disease in 2000 (49.6%), and the proportion with metastatic disease decreased from 51.6 to 37.6% over the same time period (data not shown).

### Cervical cancer

The significant decline in cervical cancer, once the most common cancer of Mumbai women, is likely due to changes in marriage and family planning, supported by underlying improvements in education and socioeconomic status. Generational changes in age at marriage and first pregnancy (National Family Health Survey India (NFHS-1) 1992–93: Maharashtra, India, 1995; National Family Health Survey India (NFHS-2): 1998–99 Maharashtra, India, 2000) have resulted in a later age at first intercourse, the primary risk factor for cervical cancer in Indian women (Prabhakar and Menon, 1995; Juneja *et al*, 2003). Higher education and socioeconomic status are associated with lower cervical cancer rates in India (Capalash and Sobti, 1999) through older age at marriage, fewer partners and pregnancies over time and through higher uptake of screening services (Shanta *et al*, 2000) and targeted cervical cancer screening and treatment interventions in rural areas have been shown to have a greater impact among women who are married, more highly educated and nulliparous (Nene *et al*, 2007). More rapid changes observed in younger birth cohorts may in part, explain the steeper declines observed in women under the age of 40 when comparing age-specific rates over time (Figure 2B).

### Ovarian cancer

Although ovarian cancer rates in Mumbai were half those in the United States in 1976 (Figure 1C), it ranked as the third most common neoplasm in Mumbai women by the year 2000 (Kavarana *et al*, 2000) and accounted for about 7% of the cancer incidence in the population. The best-fitting APC model for ovarian cancer had significant period effects with no significant change in rates over time. In Mumbai, diagnostic testing tends to be centralised in a few large hospitals (Kavarana *et al*, 2000), so the introduction of new equipment may have a more immediate impact on incidence rates, which may explain the observed increase in rates from the mid-1980s to mid-1990s across all age groups (Figure 2C). However, the subsequent decline in rates, across most age groups suggests that this factor did not have a large role; moreover, our data did not yield earlier stages at diagnosis over time. Unlike breast cancer, the clinical extent of disease remained stable (49.5% with localised disease in 1976 and 49.7% in 2000; data not shown) and the proportion of women with metastatic ovarian cancer at diagnosis did not change over the 30-year period (37.8% in 1976 and 38.5% in 2000; data not shown). And the 5-year survival of ovarian cancer patients in Mumbai remains low at 25.4% (Yeole *et al*, 2004), considerably lower than the proportions observed in the United States, Europe and other Asian countries. It is unclear that hormonal risk factors, such as age at menarche or menopause changed over time and the prevalence of other risk factors such as cigarette smoking and exogenous hormone use have remained relatively low in Mumbai women – only 1.1% of women 35 years and older reported smoking in a 1991–1992 baseline survey of a tobacco-associated cohort (Pednekar *et al*, 2006) while 2.5% were current users of oral contraceptives in 1999 (National Family Health Survey India (NFHS-2): 1998–99 Maharashtra, India, 2000).

### APC models

The APC models yielded significant non-linear cohort effects for underlying changes in breast and cervical cancer risk, which is consistent with the adoption of modern reproductive practices. Lifestyle patterns of Mumbai females changed considerably during the study period; women in Mumbai attained higher levels of education, postponed marriage, had their first child at an older age and had fewer pregnancies over time (National Family Health Survey India (NFHS-1) 1992–93: Maharashtra, India, 1995; National Family Health Survey India (NFHS-2): 1998–99 Maharashtra, India, 2000). Furthermore, India's economic developments have led to changes in diet and anthropometrics (World Bank. World development report, 1993), particularly for the higher socioeconomic classes; higher-income Indian women had 32% of their total energy from fat (Shetty, 2002) and college-educated Mumbai females had a 90% increased risk for overweight compared with illiterate women (95% CI: 1.64, 2.20) (Shukla *et al*, 2002). Both dietary fat and high body mass index are important risk factors for pre- and postmenopausal breast cancer (Wu *et al*, 1999; Key *et al*, 2003).

A graphical display of the APC parameters was included in this study, although a meaningful ‘solution’ – one that numerically allocates the drift to period and cohort on the basis of *a priori* epidemiological or biological knowledge – was beyond our present understanding of the trends, and instead we assumed a period slope of zero, allowing for the drift to be included in birth cohort. This method still allows non-linear effects for period to be visible (Holford, 1991). There may be some latency effects whereby cancers will become clinically manifest as time accrues – the effects of changing lifestyle and reproductive patterns including later age at first birth, fewer children and a higher prevalence of obesity in Mumbai women in any case may take one or more decades before they manifest themselves clinically, especially in the older age groups. And yet, the APC models detected significant effects, suggesting the method is adequate for detecting important factors in the complex trends of these three diseases.

## Conclusions

Overall, trends in breast cancer and cervical cancer incidence rates in Mumbai, India mirrored those observed in Western countries over the same 30-year time period. The changing risk profile among younger generations allied to a westernisation of lifestyle – improved education, higher socioeconomic status, later age at marriage and at first child, and lower parity – explain part of the generational changes that yielded significant changes for both cervical and breast cancer rates over time.

## Figures and Tables

**Table 1:** Number of cases and age-adjusted incidence rates for females in Mumbai, India 1976–1980 and 2001–2005 (average annual population at risk, 1.01 and 2.12 million, respectively)

	**1976–1980**			**2001–2005**			**1976–2005**	**1991–2005**
**Cancer**	**New cases** a	**% Total** b	**ASR** c	**New cases** a	**% Total** b	**ASR** c	**EAPC+95% CI (net drift)**	**EAPC+95% CI (net drift)**
Breast	331	21	39.5	1001	32	55.2	1.1 (1.0 to 1.3)	0.5 (0.0 to 1.0)
Cervix	348	23	41.1	483	16	26.6	−1.8 (−2.0 to −1.6)	−2.8 (−3.2 to –2.3)
Ovary	101	7	12.2	217	7	12.2	0.3 (−0.1 to 0.6)	−1.4 (−2.2 to −0.5)

Abbreviations: ASR=age-standardised incidence rate; EAPC=estimated annual percentage changes based on the drift; 95% CI=95% confidence interval.

a Mean annual incidence (among females aged 30–64 years).

b % Of total cancer incidence (among females aged 30–64 years).

c Truncated (among females aged 30–64 years) ASRs (World standard).

The estimated annual percent change (EAPC) for the whole period (1976–2005) and more recent period (1991–2005) are also given.

**Table 2:** Analysis of deviance of age–period–cohort models: overall goodness-of-fit and significance of model effects by cancer, trends in incidence in Mumbai 1976–2005

		**Goodness-of-fit**			**Difference between models**				
**Model no.**	**Model description**	**Deviance**	**Df** a	***P*-value**	**Model comparison**	**Model effect tested**	**Deviance**	**Df** b	***P*-value**
*Breast cancer*									
0	Age	266.4	35	<0.01			—	—	—
1	Age+drift	101.8	34	<0.01	1 *vs* 0	Drift	164.6	1	<0.01
2	Age+period	56.4	30	<0.01	2 *vs* 1	NL period	45.4	4	<0.01
3	Age+cohort	56.1	24	<0.01	3 *vs* 1	NL cohort	45.7	10	<0.01
4	Age+period+cohort^a^	28.2	20	0.10	4 *vs* 3	NL period	27.9	4	<0.01
					4 *vs* 2	NL cohort	28.2	10	<0.01
									
*Cervical cancer*									
0	Age	441.5	35	<0.01			—	—	—
1	Age+drift	100.3	34	<0.01	1 *vs* 0	Drift	341.2	1	<0.01
2	Age+period	71	30	<0.01	2 *vs* 1	NL period	29.2	4	<0.01
3	Age+cohort	44.3	24	0.01	3 *vs* 1	NL cohort	56	10	<0.01
4	Age+period+cohort^a^	34.4	20	0.02	4 *vs* 3	NL period	9.9	4	0.04
					4 *vs* 2	NL cohort	36.7	10	<0.01
									
*Ovarian cancer*									
0	Age	45.7	35	0.11			—	—	—
1	Age+drift	43.5	34	0.13	1 *vs* 0	Drift	2.2	1	0.14
2	Age+period^a^	21.0	30	0.89	2 *vs* 1	NL period	22.5	4	<0.01
3	Age+cohort	30.4	24	0.17	3 *vs* 1	NL cohort	13.1	10	0.22
4	Age+period+cohort	9.4	20	0.98	4 *vs* 3	NL period	21.0	4	<0.01
					4 *vs* 2	NL cohort	11.6	10	0.31

Abbreviation: NL=non-linear.

a Best-fitting model on grounds of parsimony.

b Degrees of freedom.
